# Biotechnological progresses in modelling the human endometrium: the evolution of current *in vitro* techniques and emerging trends

**DOI:** 10.3389/fbioe.2024.1495338

**Published:** 2024-12-03

**Authors:** Marcos Agustina-Hernández, Emilio Francés-Herrero, María Gómez-Álvarez, Paula Alonso-Frías, Mónica Romeu, Ana Monzó, Hortensia Ferrero, Clara Bueno-Fernandez, Irene Cervelló

**Affiliations:** ^1^ IVIRMA Global Research Alliance, IVI Foundation, Instituto de Investigación Sanitaria La Fe (IIS La Fe), Valencia, Spain; ^2^ Assisted Human Reproduction Unit, La Fe University and Polytechnic Hospital, Valencia, Spain; ^3^ Department of Pediatrics, Obstetrics and Gynecology, Faculty of Medicine, University of Valencia, Valencia, Spain

**Keywords:** female reproduction, endometrial modelling, *in vitro* techniques, endometrial diseases, cell culture, tissue engineering

## Abstract

The endometrium plays a fundamental role in the reproductive system yet many etiologies of infertility-related endometrial diseases such as endometriosis, adenomyosis, Asherman’s syndrome or endometrial cancer remain unknown. There are currently no treatments that minimize the effects of this devastating disorder. Appropriate model systems that closely mimic the architecture and function of the endometrium in healthy and pathological states are needed to understand the underlying molecular pathways and develop novel or more effective treatments. This review summarizes the key milestones of *in vitro* culture models of the human endometrium throughout history, as well as the applications of advanced bioengineering techniques in the modelling of both healthy and pathological endometrium. Opportunities for future approaches are also discussed.

## 1 Introduction

The human endometrium is a dynamic, hormonally-responsive tissue lining the inner cavity of the uterus. It plays a critical role in reproductive processes, particularly during menstruation, embryo implantation, and the maintenance of pregnancy. The endometrium has exceptional remodelling capacity, with monthly cycles of growth (proliferative phase), differentiation (secretory phase), degeneration (menstrual phase) and regeneration at the start of each new cycle (Maenhoudt et al., 2022).

**FIGURE 1:**
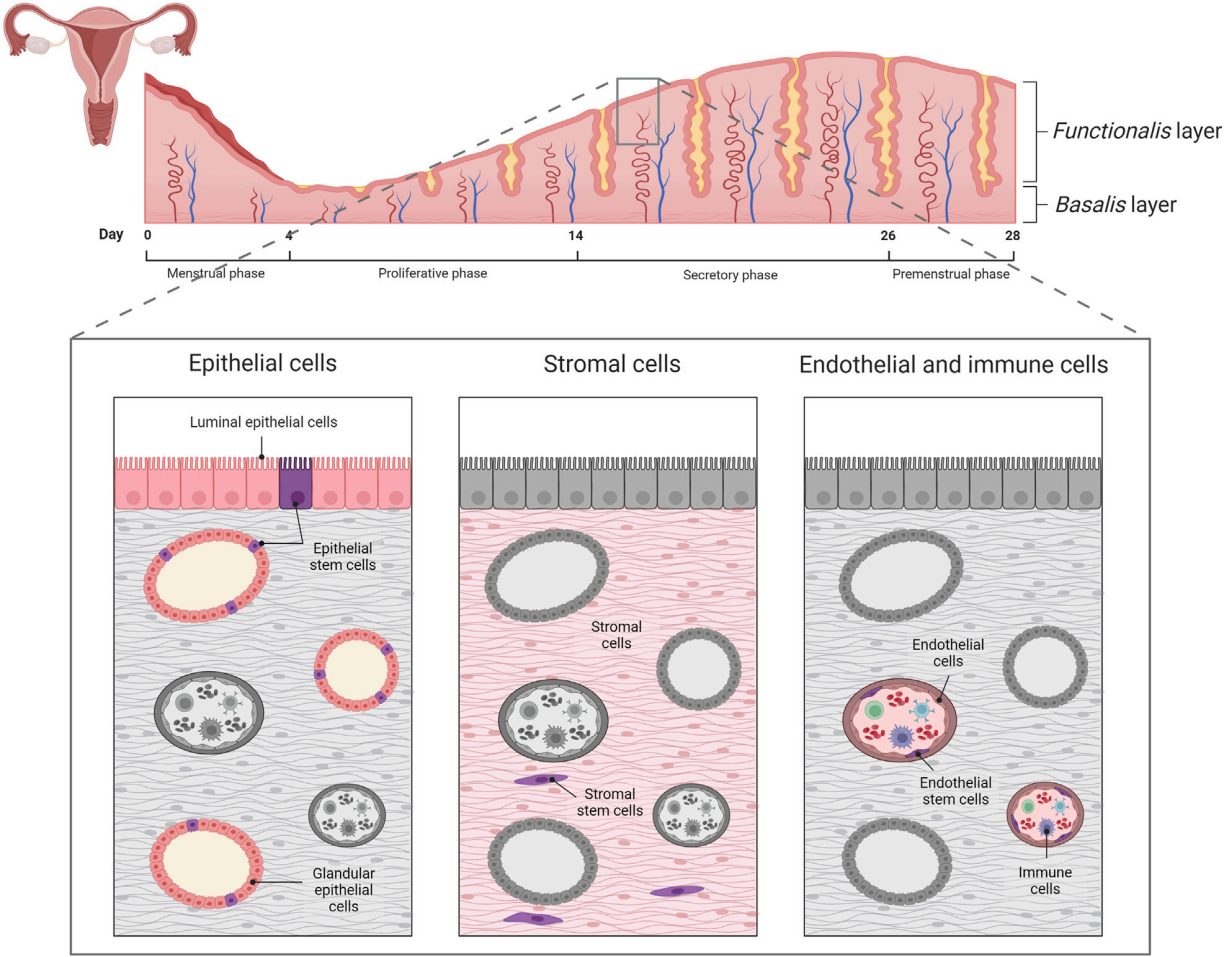
Representation of the human endometrial cycle, the layers that form it and endometrial cell populations; epithelial, stromal, and endothelial and immune cells. Created with BioRender.com.

Macroscopically, the human endometrium consists of two layers, the functionalis and basalis (Jabbour et al., 2006; Qin et al., 2024) (Figure 1). The functionalis layer, proximal to the uterine lumen, contains epithelial glands embedded in the stromal compartment and renews with each menstrual cycle. Meanwhile, the basalis layer lining the myometrium includes epithelial branching glands along with their endings, dense stroma, and large blood vessels (Maenhoudt et al., 2022; Murphy et al., 2022).

At the microscopic level, the endometrium is constituted of multiple cell types (Boretto et al., 2017) (Figure 1). The epithelial component includes both the superficial luminal epithelium and glandular epithelial cells. These cells are involved in endometrial regeneration and differentiation, supporting the cyclical growth and shedding (Cooke et al., 2013; Chemerinski et al., 2024). In contrast, stromal cells provide structural support while regulating the hormonal and vascular processes within the functionalis and basalis layers.

In addition to the epithelial and stromal compartments, the endometrial tissue hosts other cell types that are essential for the structure and function of the endometrium (Figure 1). Endometrial immune cells such as macrophages, lymphocytes and dendritic cells play key roles in immune surveillance and regulation by maintaining a tight balance between tolerance and protection (Jain et al., 2022). These cells are actively involved in the local inflammatory responses during menstruation (Vallvé-Juanico et al., 2019). Meanwhile, vascular cells, including endothelial cells and pericytes, maintain blood vessel integrity and regulate blood flow throughout the endometrial tissue. After menstruation, the endometrial vascular network provides the essential nutrients plus hormonal cues to regenerate and thicken the endometrium (Kohnen et al., 2000).

Notably, there are distinct lineages of stem cell niches within the human endometrium (Figure 1). Endometrial stem cells act as reservoirs for cellular renewal and are responsible for the endometrium’s remarkable regenerative capacity (de Miguel-Gómez et al., 2021). Endometrial stromal stem cells are located adjacent to the endothelial cells lining the microvessels in both the functionalis and basalis layers (Schwab and Gargett, 2007), whereas endometrial epithelial stem cells are exclusively located at the base of the basalis layer glands (Nguyen et al., 2017). Further, endothelial stem cells are distributed not only within the basalis layer, but also within the vascular endothelium (Mints et al., 2008; Saad-Naguib et al., 2023).

Traditional *in vitro* models used to elucidate endometrial function could not fully reflect the cellular heterogeneity or complexity of the endometrial microenvironment. Similarly, approaches to understand the biology and pathology of the human endometrium predominantly relied on *in vivo* animal studies, despite the significant biological differences with the human endometrium (Maenhoudt et al., 2022; Murphy et al., 2022). These gaps underscored the critical need to develop complex *in vitro* models capable of faithfully replicating the biological and functional native characteristics and regulatory cues of the human endometrium.

In this context, advanced *in vitro* reproductive bioengineering approaches have not only reduced the need for experimental animal models but also facilitated personalized medicine. By accurately replicating the growth, differentiation, and regeneration cycles of the human endometrium, these models are ideal for studying the underlying molecular and cellular mechanisms of endometrial development and function (Murphy et al., 2022). In addition, these techniques can be tailored to the unique characteristics of each patient, providing specific and effective therapeutic approaches for endometrial disorders, and ultimately, helping clinicians improve patients’ reproductive outcomes.

This review provides for the first time a detailed account of the most important milestones that have been achieved throughout the history of *in vitro* culture models of the human endometrium and offers a futuristic view of new bioengineering strategies.

## 2 Search methods

A search of *in vitro* cell culture milestones available in PubMed and Google Scholar was conducted by MA-H and CB-F. The search was limited to full-text articles published in English until September 2024 looking for the first paper of any important event. The following keywords were applied: human, uterus, endometrium, bioengineering, stem cells, biomaterials, microfluidic, bioprinting, organoids, hydrogel, scaffold, co-culture, cell line, extracellular matrix and endometrial populations. When the full texts were not available, a request was sent to the corresponding author(s). Additional studies were identified by manually searching the references of selected articles and complementary reviews.

## 3 The evolution of *in vitro* endometrial models

### 3.1 History of *in vitro* cell culture systems

**FIGURE 2:**
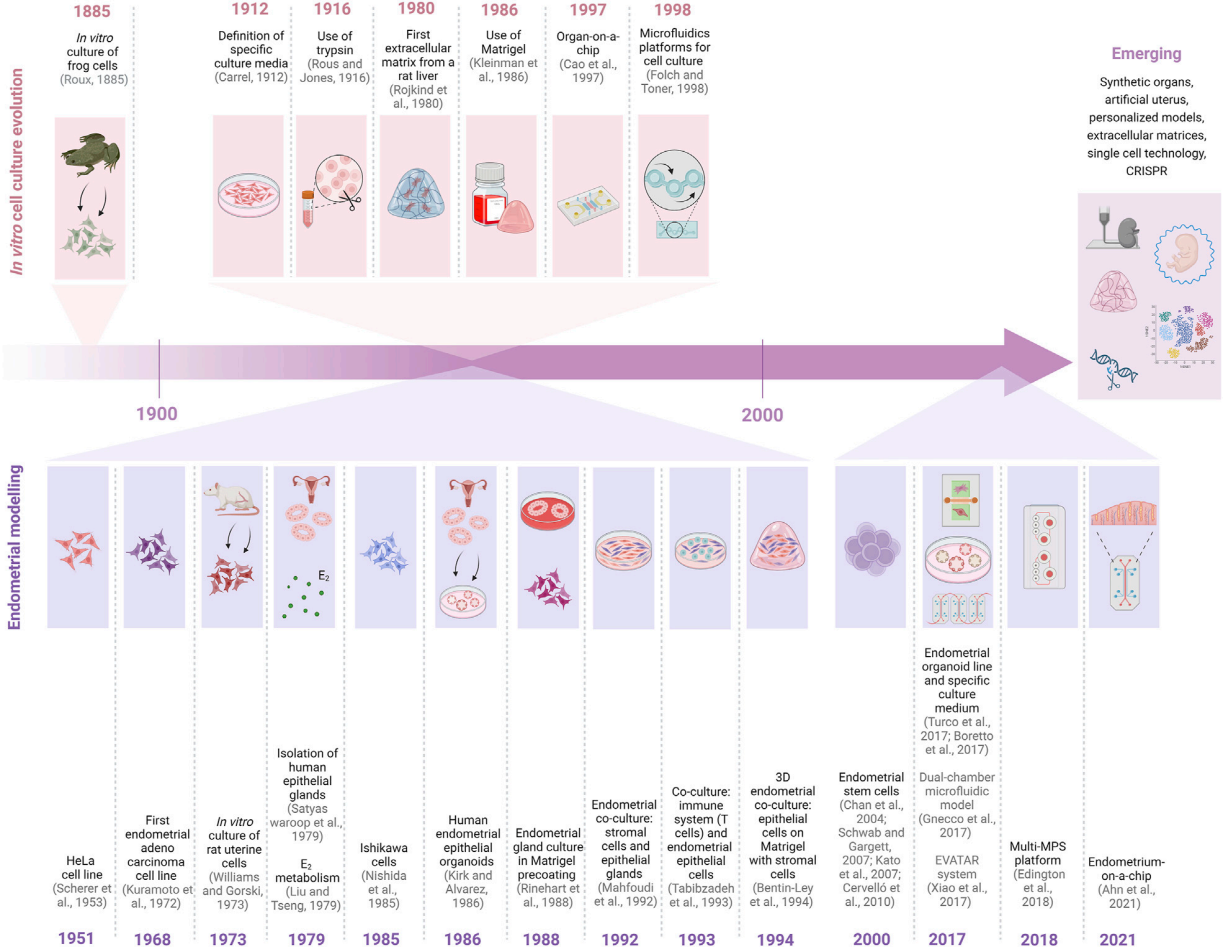
Timeline on the evolution of cell cultures techniques (depicted in the top part of the figure in pink) and advances of these techniques in the study and modelling of the endometrium (depicted in the lower part of the figure in purple). 3D, three-dimensional; CRISPR, Clustered Regularly Interspaced Short Palindromic Repeats; E_2_, estrogens; MPS, Microphysiological systems. Created with BioRender.com.

The need to recapitulate both healthy and pathological states of human tissues *in vitro* is not unique to the field of female reproduction. Throughout history, many researchers focused their efforts on developing techniques to maintain cells *in vitro* (Figure 2). It was not until 1885 that Wilhelm Roux succeeded for the first time at maintaining frog cells in the laboratory. More than 20 years later (1912), Alexis Carrel defined a specific culture medium that grew and maintained somatic mammalian cells *in vitro* for several months (Carrel, 1912; Carrel and Baker, 1926). Shortly thereafter (1916), cell culture specialists discovered that trypsin could be used to isolate individual cells from subcultures (Rous and Jones, 1916).

More than 60 years later, in the 1980s, the scientific interest shifted beyond the ability to simply grow cells *in vitro*, as the role of the cell’s native microenvironment was revealed and bioengineering techniques were being developed. This environment was considered essential for good cell growth and development. Alongside emerging bioengineering strategies to improve *in vitro* cultures, in 1980 the first *ex vivo* extracellular matrix (ECM), derived from a rat liver, demonstrated high adhesion capacity, survival efficiency and cell culture growth (Rojkind et al., 1980).

Bioengineering is a multidisciplinary field combining principles and methods from engineering, biology, and medicine to develop technologies and devices that improve healthcare and solve biological problems as tissue regeneration or development of medical devices (Francés-Herrero et al., 2022). The use of bioengineering was a turning point in the approach of cell cultures to the native environment. In that sense, in 1986, the description of the use of gel-like extracellular matrix components as a suitable microenvironment for cell growth was published for the first time (Kleinman et al., 1986). The subsequent commercialization of this protein-rich matrix, coined Matrigel (BD Biosciences, formerly BD Pharmingen), was a significant research milestone because it supported three-dimensional (3D) *in vitro* models that reliably mimicked the native conditions that promote cell differentiation and growth, among other advantages.

Advanced *in vitro* bioengineering approaches became popular at the end of the 20^th^ century. Microfluidic devices were designed to mimic the endocrine and paracrine signalling between different tissues of human organs. Microfluidic devices combine principles from engineering, physics, chemistry, biology, and nanotechnology for highly specialized and efficient applications. For example, the first organ-on-a-chip dates back to 1997 and it involved chondrocytes on a polyglycolic acid polymer scaffold. They introduced these combination and helped to form cartilage 12 weeks post-implantation in mice (Cao et al., 1997). Finally, microfluidic platforms for *in vitro* cell cultures were first reported in 1998. Folch and Toner injected collagen- or fibronectin-rich protein solutions through microchannels to create a biocompatible scaffold to seed different cell populations such as fibroblasts (Folch and Toner, 1998).

### 3.2 Endometrial modelling

The most revolutionary milestone in endometrial (and other tissues) cell culture was the establishment of continuous cell lines. This was important because healthy animal-derived somatic cells have a limited proliferative capacity. An important paradigm shift in *in vitro* culture practices began in 1951, when continuous human cell lines were established. The laboratory of George and Margaret O. Gey established a patient-derived monolayer cell culture from immortalized cervical cancer cells. The resulting HeLa cell line, named after the original patient from whom the primary cells were derived, Henrietta Lacks (Scherer et al., 1953), has become one of the oldest and most commonly used cell lines across the globe. The development of stable cell lines was crucial, as it allowed for homogenizing the cell population and did not require sacrificing any animals. HeLa cell cultures paved the way for more sophisticated monolayer cell culture techniques and helped characterize additional cell lines derived from reproductive tissues. Specifically, the human endometrial adenocarcinoma cell line (HEC-1) was characterized and immortalized in 1968 (Kuramoto et al., 1972). The HEC-1 cell line was mainly used to study the endometrium and endometrial carcinoma in a simplified cellular system (Kurarmoto et al., 2002). Following, in 1973, for the first time, viable uterine cells were directly obtained from a rat uterus. The authors defined the metabolic characterization of these cells and revealed the equilibrium dissociation constant for estrogen binding (Williams and Gorski, 1973), the guidelines for isolating and maintaining human endometrial epithelial glands *in vitro* were established in 1979 (Satyaswaroop et al., 1979). For the first time, these guidelines recommended that endometrial glands be isolated by enzymatic digestion with collagenase and cultured with a nutrient-rich media (Satyaswaroop et al., 1979). This approach led to the co-culture of primary human endometrial epithelial glands with stromal cells to study estradiol metabolism during the menstrual cycle (Liu and Tseng, 1979).

Five years later, in 1985, the Ishikawa cell line was derived from a stage II human endometrial adenocarcinoma (Nishida et al., 1985). Clones of these cells are still available to researchers across the globe, with varying expression of estrogen receptors (ERs) and progesterone receptors (PRs) depending on the clone’s degree of differentiation (Nishida, 2002). Nevertheless, it remains difficult to study human endometrial epithelial cells’ hormonal responses in monolayer cultures, as they do not accurately reflect the complexity of the uterine microenvironment. A 3D cellular arrangement was found to be necessary to maintain the cells’ differentiation state which is related to hormone sensitivity.

In 1986, Kirk and Alvarez developed a new method for *in vitro* culture of reproductive tissues using Matrigel, in which cells maintain their 3D morphology (Kirk and Alvarez, 1986). This technique is considered to have generated the first epithelial organoids from human endometrial biopsies. Small fragments of endometrial tissue were processed through gravity sedimentation to isolate the smallest glandular elements, which, in turn, were cultured with a defined media supplemented with a collagen gel (Kirk and Alvarez, 1986). Alternatively, in 1988, biopsy-derived endometrial gland elements that were cultured on a Matrigel coating for several weeks produced larger colonies, with 50% of the elements assembling into large organoid structures (Rinehart et al., 1988). Notably, epithelial cells grown on traditional plastic culture plates remained flat and squamoid whereas the cells grown on Matrigel adopted their natural columnar shape. Further, the ECM support from the Matrigel consistently provided longer periods of cell growth and maintenance compared to traditional plastic plates (3 months vs. 3 weeks, respectively). Finally, it was also in 1988 when another important event in the field of endometrial modelling took place. The hormone-responsive endometrial cell line (ECC1) was cloned from human endometrial adenocarcinoma cells (EnCa101AE) transplanted into mice (Tabibzadeh et al., 1988). To date, these cells have been widely used to study the endometrium’s hormone response, as they express ER and PR.

In the early 1990s, researchers focused on co-cultures to elucidate the interactions between different cell populations in the female reproductive system. The first experimental article combining endometrial epithelial glands cultured over a Matrigel coating with stromal cell monolayers was published in 1992. This co-culture modelled the native paracrine interactions, improving the understanding of endometrial function (Mahfoudi et al., 1992). The following year (1993), Tabibzadeh’s group demonstrated that the immune system (T cells) regulates the activity of endometrial epithelial cells. The presence of T cells in ECC1 cultures altered antigen expression and proliferation rates (Tabibzadeh et al., 1993), highlighting the role of the microenvironment. Next, to add another layer of complexity to endometrial models, in 1994, Bertin-Ley’s group co-cultured endometrial epithelial cells grown on a Matrigel membrane with endometrial stromal cells embedded in a collagen matrix. This 3D model faithfully recapitulated the natural interactions between the stromal compartment (composed of ECM and stromal cells) and epithelial compartment, providing a reliable model of the human endometrium *in vivo* (Bentin-Ley et al., 1994).

In the 2000s, independent research groups corroborated the existence of human endometrial stem cells. A cohort of epithelial and stromal cells with clonogenic activity was first observed in the human endometrium in 2004 (Chan et al., 2004). Three years later, Schwab and Gargett published a methodology for isolating the subpopulation of human endometrial stromal cells with mesenchymal stem cell colony-forming and multipotent properties (Schwab and Gargett, 2007). Finally, in 2007, Kato et al. published the isolation of the Side Population (endometrial stem cells that can participate in the regeneration of the tissue and have self-renewal potential), confirmed 3 years later by Kato et al. (2007), Cervelló et al. (2010). These findings have had wider implications in the field of reproduction such as the understanding of the mechanisms of endometrial regeneration and their role in this process.

Around 15 years later, Turco et al. established endometrial epithelial organoid models in 2017. In contrast to the endometrial organoids from 1986, these novel structures could be used for long-term cultures and preserved their genetic stability plus high proliferation rates even after freeze-thaw cycles. Turco’s group defined a culture medium for maintaining and expanding endometrial epithelial organoids as well as a method for differentiating these organoids into a secretory or gestational state (Turco et al., 2017). Meanwhile, Boretto et al. characterized expandable healthy and pathological (endometrial cancer) human endometrial organoid models (Boretto et al., 2017). Together, these advances created the foundation for patient-specific biobanks that could be used for personalized drug testing, among other applications.

The first approach of microfluidic models for the human endometrium emerged in 2017. Gnecco et al. developed a dual-chamber microfluidic platform co-culturing primary human endothelial and endometrial stromal cells to mimic the physiological changes occurring throughout the 28-day human menstrual cycle. The device consisted of two orthogonal microfluidic chambers, made with polydimethylsiloxane, divided by a biocompatible resin-based membrane permitting cell communication via soluble factors. This system allowed the simultaneous analysis of stromal decidualization and endometrial vascular function under controlled physiomimetic conditions, such as endothelial cells remodelling and vascular barrier formation (Gnecco et al., 2017). In 2017, Xiao et al. developed the EVATAR system to co-culture murine ovaries with human fallopian tube, endometrium, ectocervix and liver tissues for 28 days. Each tissue was cultured in its own chamber and connected by microfluidic channels with dynamic media flow to effectively recapitulate pituitary hormone circulation and regulation (Xiao et al., 2017). Next year, Edington et al. described a 10-way multi-microphysiological system platform including the liver, gut, lung, heart, pancreas, brain, skin, kidney, skeletal muscle and endometrium. This system replicates the functional interactions between multiple human tissues in a laboratory setting and offers a more precise approach to studying human biology and pharmacology (Edington et al., 2018).

Finally, in 2021, Ahn et al. recently developed a micro-engineered vascularized endometrium-on-a-chip (MVEOC) which reconstituted the physiologically relevant endometrial environment, containing the three main cell populations of the human endometrium. By combining endothelial, epithelial and stromal cells, this device serves as a promising model to study endometrial angiogenesis and vasculogenesis, among other biological processes. Notably, the MVEOC can improve efficiency of high-throughput drug screening and identify molecular pathways involved in the process of embryo implantation (Ahn et al., 2021).

In the near future, novel techniques have emerged such as synthetic or artificial organ development and bioprinting, which allow the creation of lab-grown tissues for regenerative medicine applications. Generating personalized models is also an alternative to test treatments in an individual’s specific biology. Tissue-specific ECMs are used in tissue regeneration and are designed to mimick the natural cellular environment. Finally, single-cell technologies and CRISPR genome editing are also emerging as new strategies, among others.

## 4 Biotechnological progresses in healthy and pathologic endometrial models

**FIGURE 3:**
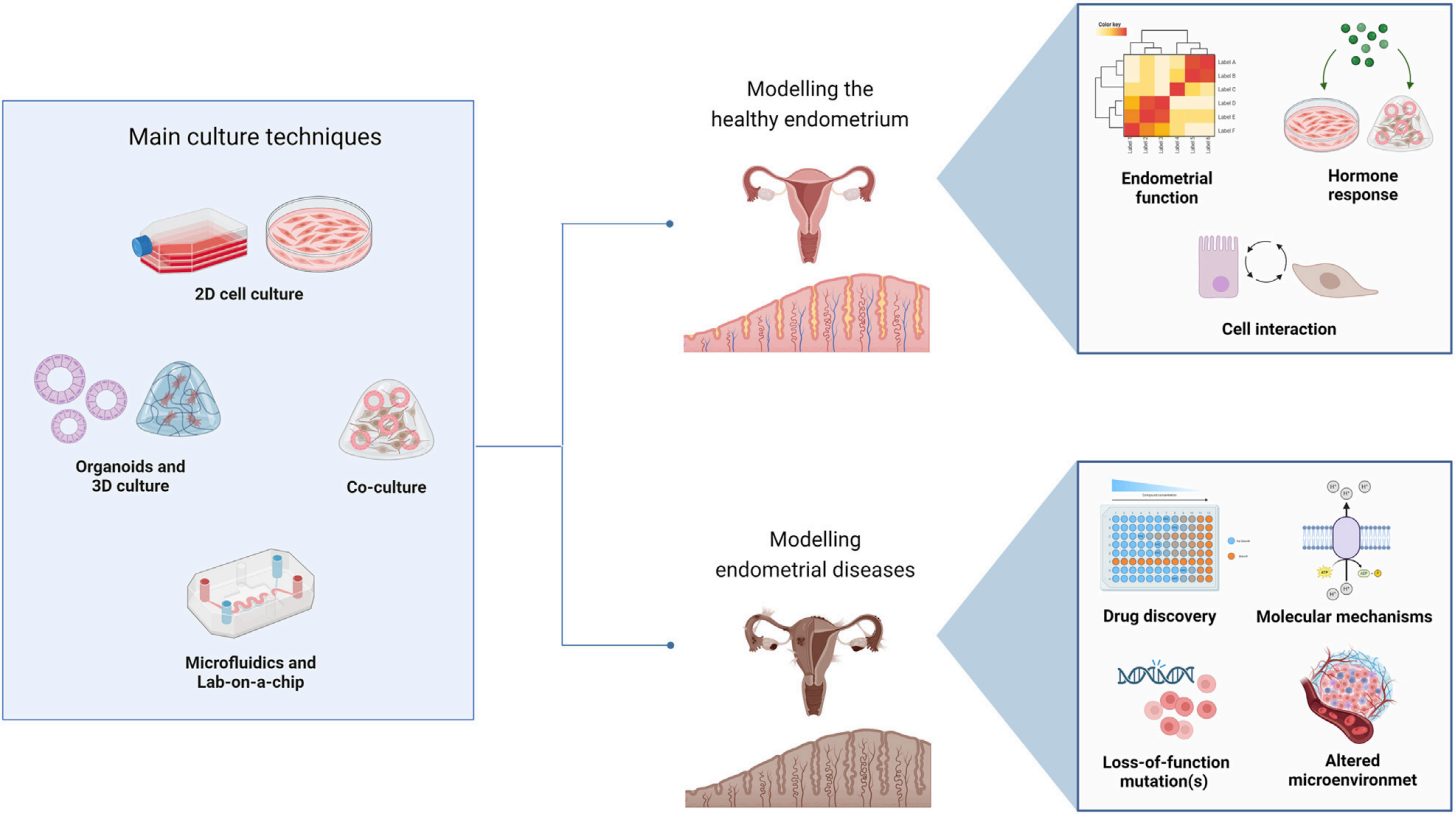
Applications of culture techniques for healthy and pathological endometrium models. Created with BioRender.com.

Biotechnology has significantly enhanced our understanding of the molecular and cellular mechanisms underlying endometrial diseases, such as endometriosis and endometrial cancer, revealing new therapeutic avenues. This section will explore biotechnological applications in endometrial research, including modelling healthy and diseased endometrium, endometrial repair, drug screening, and therapeutic drug monitoring (Figure 3).

### 4.1 Modelling healthy endometrium states

Cell culture techniques have been instrumental for studying the physiology of the healthy endometrium. Primary endometrial cells, stem cells and cell lines provide valuable models for investigating endometrial function, hormone responses, and cell interactions (Pijnenborg et al., 2023). These models are used to explore the endometrium’s cyclical changes and response to hormone therapy. Although 2D cell cultures have dominated the field for a long time, recent research has shifted toward culturing 3D structures as they provide enhanced biomimicry in terms of structure and physiology. Specifically, endometrial organoid models closely mimic the structural and functional characteristics of the native endometrial tissue, accurately recapitulating the conditions of the *in vivo* microenvironment (Boretto et al., 2017). Among many other applications, organoids are used to study endometrial regeneration, hormone regulation, and the interactions between endometrial and immune cells (Dai et al., 2024). Finally, the microfluidic devices and lab-on-a-chip technologies represent dynamic platforms to model the endometrium. These systems can simulate the menstrual cycle, among other physiological processes, by controlling the microenvironment and fluid flow around endometrial cells (De Bem et al., 2021). Such models are particularly useful for studying cell signaling, tissue dynamics, and the effects of pharmacological agents in a controlled setting (Murphy et al., 2022).

### 4.2 Modelling pathological endometrium states

Models of endometrial diseases, particularly endometriosis or adenomyosis, are generated through various *in vitro* and *in vivo* approaches. *In vivo* rodent models are particularly valuable for studying the pathogenesis of endometriosis and testing new therapeutic approaches (Burns et al., 2021). Alternatively, *in vitro* endometrial organoid models provide insights into the molecular mechanisms driving disease initiation and progression (Luddi et al., 2020; Esfandiari et al., 2021a; Esfandiari et al., 2021b). Adenomyosis, has also been studied using diverse *in vitro* models. Notably, 3D cultures and organoid models were used to study the invasive behavior of endometrial cells along with their interactions with adjacent myometrial tissue (Mehasseb et al., 2010; Taylor et al., 2014). Endometrial cells co-cultured with immune cells or bone marrow-derived stem cells helped elucidate the complex cell interactions in endometriosis (Chan et al., 2017; Chen et al., 2021). Finally, microfluidic devices were used to mimic the excessively active uterine peristalsis (hyperperistalsis) driving development of adenomyosis or endometriosis. By simulating the dynamic microenvironment of the uterus, researchers were able to study the kinetics of cellular migration and invasion (Elad et al., 2020).

Endometrial cancer research has significantly benefited from biotechnological advancements. Cancer cell lines, patient-derived xenografts, and genetically engineered mouse models are used to study tumor biology and progression, identify genetic mutations, and evaluate potential treatments (Dai et al., 2024). These models have been instrumental in understanding the role of specific oncogenes and tumor suppressors in endometrial cancer development (Hibaoui and Feki, 2020). Specifically, organoids derived from endometrial cancer patients were developed to study the tumor microenvironment and test the efficacy of targeted and personalized therapies (Berg et al., 2021).

In summary, recent biotechnological approaches facilitated the development of high-throughput drug screening as well as monitoring of endometrial disease models. Automated systems accelerate evaluation of drug efficacy and toxicity in endometrial organoids (Girda et al., 2017), streamlining identification of new therapeutic compounds. Further, multiple drug candidates can simultaneously be tested on patient-derived organoids, providing personalized insights into how particular patient could respond to treatment, thereby helping clinicians optimize treatment regimens (Boretto et al., 2019). Further, live-cell imaging and biosensors can be used to monitor the dynamics of cellular responses to treatments in real-time allowing early detection of a treatment’s efficacy or toxicity before adverse effects occur (Peel and Jackman, 2021).

## 5 Emerging trends and future perspectives

Cutting-edge bioengineering technologies and innovative study approaches are driving promising advances in endometrial research. Novel materials and techniques are being leveraged to replicate the native human endometrium *in vitro*. Approaches based on organoids, microfluidic systems, hydrogels, and decellularized ECM scaffolds are elucidating the fundamental mechanisms of reproductive biology and pathogenesis. By streamlining drug discovery for endometrial diseases, these platforms have the potential to significantly enhance reproductive function and fertility (Murphy et al., 2022; Dai et al., 2024).

Despite advances in *in vitro* endometrial models, significant limitations and challenges remain unsolved (Fitzgerald et al., 2021). First, while 2D models are accessible and useful, they fail to replicate the structural and functional complexity of the endometrial microenvironment, limiting their ability to predict precise cellular responses to hormonal or drug stimuli. Although 3D models and organoids offer improved biomimicry, they exhibit variability in standardization and consistency in reproducing the physiological conditions of endometrial tissue (Gómez-Álvarez et al., 2023). Lab-on-a-chip technology and microfluidic devices simulate dynamics closer to the *in vivo* environment; however, their implementation remains costly and technically complex, which may hinder their use in high-throughput studies. Additionally, current models still face challenges in fully capturing the cellular interactions within the uterine microenvironment, such as those between immune and endometrial cells, which are crucial for understanding complex pathologies like endometriosis or endometrial cancer (Murphy et al., 2022).

One of the main ongoing challenges in endometrial research is developing comprehensive models that accurately recapitulate the multi-faceted genetic, epigenetic, and molecular relationships along with the native endometrial characteristics. Although significant progress has been made, current human endometrial models have not fully encompassed the complexities of endometrial biology and associated diseases. Standardizing substrates for *in vitro* culture systems with bio-scaffold systems such as hydrogels, decellularized scaffolds, and microfluidics, will be crucial for addressing this gap. These customizable and versatile solutions for organoid preparation help tailor culture environments to specific *in vivo* conditions (Francés-Herrero et al., 2022).

Innovative approaches combining endometrial organoids with hydrogels are promising to study endometrial pathologies, including endometriosis, endometrial cancer, intrauterine adhesions, and the decidualization process. These approaches help bridge the gap between *in vitro* culture systems and native tissues, offering tunable properties and improved therapeutic benefits in regenerative medicine (Dai et al., 2024). Decellularized endometrium scaffolds provide a bioactive microenvironment that enhances organoids’ proliferative properties and chromosomal stability. Culturing endometrial organoids with bio-scaffolds replicates the *in vivo* biochemical signals, which is critical for studying disease mechanisms and therapeutic responses (Francés-Herrero et al., 2021; Gómez-Álvarez et al., 2024).

Microfluidic systems offer significant advances over traditional static cultures by incorporating mechanical flow dynamics, continuously replenishing media while eliminating toxic metabolites. Nevertheless, the clinical utility of microfluidic technology can be improved by fabricating the platforms out of materials that do not absorb sex steroid hormones and integrate biosensors for real-time data collection. Alternatively, microfluidic platforms that can support extended culture periods may be employed to model sequential menstrual cycles, providing valuable insights into how molecular alterations in the endometrium accumulate over time due to risk factors like unopposed estrogen action (Campo et al., 2023).

Three-dimensional bioprinting is a potential innovative culture technique in endometrial modelling by precisely replicating tissue architecture and enhancing cellular function, in contrast with organ on a chip models. Recent studies demonstrate that 3D bioprinted hydrogel scaffolds loaded with stem cells or biomimetic constructs significantly improve endometrial repair (Ji et al., 2020). For instance, using stem cell-loaded scaffolds has been shown to enhance cellular regeneration, histomorphology, and endometrial receptivity, partially restoring the implantation and pregnancy functions in damaged tissues (Ji et al., 2020). Additionally, a bilayer construct mimicking the native structure of the endometrium efficiently restores tissue morphology and improves significantly reproductive outcomes in severe injury models (Nie et al., 2023). Moreover, integrating 3D bioprinting with sustained-release systems for targeted drug delivery, such as G-CSF-loaded microspheres, enhances specific treatments, reduces fibrosis, and promotes vascular and cell regeneration (Wen et al., 2022). All these approaches underscore the potential of 3D bioprinting as an advanced and individualized strategy for structural and functional endometrial repair, offering new treatments for patients with infertility due to endometrial damage.

Artificial intelligence (AI) is transforming the study of the human endometrium, especially in the context of endometrial diseases, by enabling complex analyses that identify biomarkers and pathological mechanisms which traditional methods struggle to detect (Rewcastle et al., 2023; Yang et al., 2024). Integrating AI into these studies provides a faster and more precise approach to simulating endometrial diseases, significantly advancing toward high-relevance preclinical models for clinical applications (Chen et al., 2023; Raimondo et al., 2024).

Tumor biopsy-derived organoids have shown great potential for drug testing and personalized treatment of endometrial cancer. These organoids maintain the genetic heterogeneity and unique histological characteristics of the original tumors and can be used to screen chemotherapy agents, predict patient-specific drug responses or explore new therapeutic strategies. Endometrial cancer organoids reliably model tumor histology and genetics, highlighting their suitability for biobanking and precision medicine applications (Berg et al., 2021; Gómez-Álvarez et al., 2023).

Through innovations like organoids, microfluidic systems, 3D bioprinting, and the application of AI, new complex strategies are being developed not only to accelerate drug discovery but also to enable the creation of more effective, patient-specific regenerative therapies. These advancements represent a critical step toward implementing personalized medicine strategies, enabling more accurate diagnoses and targeted treatments that enhance reproductive health and improve the quality of life for patients affected by endometrial diseases.

## 6 Major findings and conclusions

The evolution of *in vitro* cellular models of the endometrium has been marked by significant milestones, beginning in 1951 with the development of HeLa cell line, which revolutionized reproductive *in vitro* research (Scherer et al., 1953). In 1968, the HEC-1 endometrial adenocarcinoma cell line facilitated the study of endometrial pathophysiology (Kuramoto et al., 1972). Starting in the 1970s, progress in enzymatic dissociation and co-culture techniques enabled investigation of endometrial function, estrogen metabolism, and cellular interactions (Liu and Tseng, 1979), while in the 1980s, 3D cultures using ECMs (such as Matrigel) provided a better replication of the uterine microenvironment (Kirk and Alvarez, 1986). Throughout the 1990s and 2000s, co-culture models and the discovery of endometrial somatic/adult stem cells (Cervelló et al., 2010; Cervelló et al., 2011; Cervelló et al., 2012) with regenerative properties further enriched understanding of the endometrium (Kato et al., 2007). In 2017, advanced organoid and microfluidic systems, including endometrium-on-a-chip models, offered new insights for modelling endometrial function and personalized drug testing (Turco et al., 2017; Ahn et al., 2021).

The study of both healthy and pathological endometrium has been increased by the advancements of the *in vitro* endometrial models. Cell culture techniques, including primary cells, stem cells, and cell lines, serve as key models for exploring endometrial physiology, hormonal responses, and cell interactions (Francés-Herrero et al., 2022). The shift towards 3D models and organoids has improved structural and functional biomimicry, enabling detailed studies of endometrial regeneration, immune interactions, and hormonal regulation. Nowadays, microfluidic and lab-on-a-chip technologies simulate dynamic endometrial processes like the menstrual cycle and cell signaling, offering controlled settings for pharmacological testing. In studying endometrial pathologies such as endometriosis, adenomyosis, and cancer, *in vitro* organoids, 3D cultures, and microfluidic devices allow researchers to investigate disease mechanisms, tumor biology, and cellular invasion. These models also facilitate high-throughput drug screening and therapeutic monitoring, particularly with patient-derived organoids for personalized treatment. Additionally, 3D bioprinting enables the precise replication of tissue architecture, improve tissue repair and endometrial regeneration, while AI accelerates the identification of biomarkers and therapeutic targets, optimizing personalized treatments and advancing clinically relevant preclinical models.

In conclusion, advanced bioengineering technologies such as organoids, microfluidic systems and bio-scaffolds have shifted the paradigms for traditional endometrial models and are significantly enhancing our understanding of human endometrial biology and pathology. These innovative *in vitro* models support study reliability, efficacy and validity, improving clinical therapies and interventions for endometrial diseases. As the research landscape continues to evolve, these technologies will play a crucial role in unraveling the complexities of the endometrium, enabling more accurate diagnoses and targeted treatments, and developing personalized treatment strategies to improve the quality of life for patients affected by endometrial diseases.
